# Exercise testing criteria to diagnose lower extremity peripheral artery disease assessed by computed-tomography angiography

**DOI:** 10.1371/journal.pone.0219082

**Published:** 2019-06-27

**Authors:** O. Stivalet, A. Paisant, D. Belabbas, L. Omarjee, A. Le Faucheur, P. Landreau, R. Garlantezec, V. Jaquinandi, D. A. Liedl, P. W. Wennberg, G. Mahé

**Affiliations:** 1 Vascular Medicine Unit, CHU Rennes, Rennes, France; 2 Vascular Medicine, CH de Saint Malo, Saint-Malo, France; 3 Radiology Department, CHU Rennes, Rennes, France; 4 Vascular Medicine Unit, CH de Redon, Redon, France; 5 Univ Rennes; INSERM CIC, Rennes, France; 6 Ecole Normale Supérieure, Bruz, France; 7 Gonda Vascular Center, Mayo Clinic, Rochester, MN, United States of Amerca; Nagoya University, JAPAN

## Abstract

**Background:**

The sensitivity and specificity of exercise testing have never been studied simultaneously against an objective quantification of arterial stenosis. Aims were to define the sensitivity and specificity of several exercise tests to detect peripheral artery disease (PAD), and to assess whether or not defined criteria defined in patients suspected of having a PAD show a difference dependent on the resting ABI.

**Methods:**

In this prospective study, consecutive patients with exertional limb pain referred to our vascular center were included. All patients had an ABI, a treadmill exercise-oximetry test, a second treadmill test (both 10% slope; 3.2km/h speed) with post-exercise pressures, and a computed-tomography-angiography (CTA). The receiver-operating-characteristic curve was used to define a cut-off point corresponding to the best area under the curve (AUC; [CI95%]) to detect arterial stenosis ≥50% as determined by the CTA.

**Results:**

Sixty-three patients (61+/-11 years-old) were included. Similar AUCs from 0.72[0.63–0.79] to 0.83[0.75–0.89] were found for the different tests in the overall population. To detect arterial stenosis ≥50%, cut-off values of ABI, post-exercise ABI, post-exercise ABI decrease, post-exercise ankle pressure decrease, and distal delta from rest oxygen pressure (DROP) index were ≤0.91, ≤0.52, ≥43%, ≥20mmHg and ≤-15mmHg, respectively (p<0.01). In the subset of patients with an ABI >0.91, cut-off values of post-exercise ABI decrease (AUC = 0.67[0.53–0.78]), and DROP (AUC = 0.67[0.53–0.78]) were ≥18.5%, and ≤-15mmHg respectively (p<0.05).

**Conclusion:**

Resting ABI is as accurate as exercise testing in patients with exertional limb pain. Specific exercise testing cut-off values should be used in patients with normal ABI to diagnose PAD.

## Introduction

Peripheral artery disease (PAD) affects more than 200 million people worldwide.[1] The ankle brachial index at rest (ABI) is the ratio of the highest systolic blood pressure measured in each ankle to the highest systolic blood pressure measured in the arms. [2,3] This is the main clinical test recommended by the guidelines of the American Heart Association (AHA) to diagnose the presence and severity of PAD regardless of the symptoms presented by patients.[4]

For intermittent claudication (exertional limb pain that is relieved by rest), it has been suggested that exercise or post-exercise assessments (ABI, Doppler waveforms analysis) could be better than resting investigations to detect stenosis that are hemodynamically silent at rest but significant during exercise. According to AHA recommendations the ABI should be the first line tool to diagnose PAD. [5] In case of normal ABI and strongly suspected of having a PAD, the AHA statement proposed two different post-exercise criteria to diagnose PAD; either a post-exercise ABI decrease > 20% or a post-exercise pressure decrease > 30mmHg. We have shown previously that these criteria are of concern for several reasons: i) they do not identify PAD the same patients [6]; ii) they were established without quantifying the degree of arterial stenosis anatomically; iii) the exercise protocols utilized in those studies are now uncommon; iv) they were defined on subjects without reference to the ABI whereas AHA stated that they should be used in patients with ABI > 0.90. It is unlikely that post-exercise findings are similar in patients suspected of having a PAD with ABI > 0.90 compared to the findings in patients regardless of the ABI. Moreover, an updated exercise protocol used for exercise-oximetry (exercise-TcPO2) has been developed.[7,8] In our previous study we have confirmed in 34 patients included from 2014 to 2015 the accuracy of exercise TcPO2 to diagnose arterial stenosis.[8] However, in this study the accuracies of the other classical tests (ABI and post-exercise ABI) were not studied. Furthermore, the accuracy of exercise TcPO2 to diagnose arterial stenosis in patients with normal ABI is unknown.

To date, no study has been performed that simultaneously defines the sensitivity and specificity of multiple exercise tests and parameters (post-exercise ABI decrease, post-exercise pressure decrease, and exercise-TcPO2) in patients with exertional-limb pain regardless of the ABI against the degree of stenosis. Therefore, the aims of this study were i) to define optimal cut-off value for exercise tests with their sensitivity and specificity to detect arterial stenosis as quantified by CTA; ii) to assess whether or not defined criteria defined in patients suspected of having a PAD show a difference dependent on the resting ABI.

## Materials and methods

### Study design and population

This is a monocentric study on consecutive patients referred to our vascular unit (University Hospital, Rennes, France) for exertional limb pain after a careful clinical examination.

The study was conducted from May 2016 to June 2017 and approved by an institutional review board from the University Hospital of Rennes (ref. 17.12). All participants gave written informed consent. The study protocol conforms to the ethical guidelines of the 1975 Declaration of Helsinki. The exercise PAD study was registered with the American National Institutes of Health database under reference n° NCT03186391.

In this population, we selected patients who had a CTA performed within 3 months of the exercise appointment. In our clinical practice, we systematically perform an exercise-TcPO2 followed by a second exercise test to measure the post-exercise ankle pressure and ABI. Patients unable to walk on the treadmill, or suffering cardiac pathology contraindicating a stress test were excluded.

### Demographic characteristics

Variables collected included age, sex, body mass index, comorbidities, and medications (statins, anti-hypertension treatment, antiplatelet, antidiabetic oral treatment or insulin).

### ABI measurement

After clinical evaluation, a baseline resting ABI was performed according to AHA recommendations [4] using a hand-held Doppler probe (8 MHz; Basic Atys Medical, Soucieu en Jarrest, France) and a 10 cm cuff by a trained vascular medicine physician at the ankle level. In brief, the patient was at rest for 10 minutes in the supine position, relaxed, head and heels supported, in a room with comfortable temperature (21°C).[9] The following counterclockwise sequence was used: right brachial artery, right posterior tibial artery, right dorsalis pedis artery, left posterior tibial artery, left dorsalis pedis artery, left brachial artery, and right brachial artery. The ABI was calculated by dividing the artery highest pressure of the limb (dorsalis pedis or posterior tibial pressures) by the highest arm pressure.[10]

For the brachial artery, contrary to the AHA guidelines, we used an automatic blood pressure monitor (Carescape Dinamap V100; GE Healthcare, Chicago, Illinois, United States of America) in order to have the same technique to measure the pressure at rest and after exercise.

### Treadmill test

A treadmill test (3.2km/h, 10% slope) was used up to a maximal distance of 1053m (20 minutes). This test was used for both the exercise-TcPO2, which was performed first, and for the post-exercise pressure measurements. A minimum of 10 minutes was required between the two exercise tests. The patients were asked to inform the physician when and where (buttock, thigh, calf or other) symptoms occurred. Exercise was stopped for both studies according to patient’s limitation.

### Exercise-TcPO2 test

Briefly, measurement of TcPO2 was performed using calibrated TcPO2 electrodes (TCOM/TcPO2; PF 6000TcPO2/CO2 Unit; Perimed; Jarfalla, Sweden) as previously described.[7,11,12] Exercise was performed on a treadmill at a 10% slope and a speed of up to 3.2km/h.[11] Exercise was discontinued at the patient’s request or, by protocol, up to maximum exercise duration of 20 minutes. A reference electrode (chest electrode) was placed between the scapulae to measure systemic changes in TcPO2 during exercise. One electrode was positioned on each buttock, 4 to 5 cm behind the bony prominence of the trochanter, and one electrode on each calf.[11] The measurements from the TcPO2 electrodes were used to calculate the delta from rest oxygen pressure (DROP) index which was expressed in mmHg and was recorded in real time by the in-house Oxymonitor Software as previously described.[12,13]

Results of exercise-TcPO2 were analyzed without knowledge of CTA results. Right distal DROP allows the detection of flow-reducing lesions in the following arteries; the aorta, the right common iliac artery, the right external iliac artery, the right common femoral artery, the right superficial femoral artery, and the right popliteal artery. Left distal DROP allows the detection of flow-reducing lesions in the following arteries; the aorta, the left common iliac artery, the left external iliac artery, the left common femoral artery, the left superficial femoral artery, and the left popliteal artery.[12] A recent study has shown that exercise-TcPO2 using a minimal DROP value is accurate to diagnose arterial stenoses of ≥ 50% assessed by computed tomography angiography (CTA) as a gold standard that confirmed previous results.[8]

### Post-exercise pressure and post-exercise ABI measurements

Two technicians performed the measurements: one at the brachial level with the automatic blood pressure device and one at the limb level with the handheld Doppler.

Post-exercise brachial pressure was assessed on the same artery as used for the ABI measurement. When the ABI was measured, a black pen was used to mark the skin area where the highest limb pressure had been recorded with a hand-held Doppler. Following exercise, we were sure that we were on the correct area to perform the post-exercise pressure measurement. If there was no arterial flow detected the pressure were recorded as 0 mmHg.[14] The highest ankle pressure of each limb at rest was assessed, beginning with the more symptomatic limb. Post-exercise pressures were assessed within 1 minute after the termination of walking.

### Arterial stenosis quantification using CTA

CTA was performed in all subjects within 3 months before or after the post-exercise ABI and exercise-TcPO2 measurements. CTA was performed with a 64-slice CT scanner (Discovery CT 750 High Definition; GE Healthcare, Milwaukee, WI, USA), 100‐kV tube voltage and an automatic modulation of mAs (80–500 mAs). The scanning range was planned with a scout view and included the entire vascular tree from the abdominal aorta to ankles. A total of 120 mL or 1.5ml/kg of iobitridol 350 mgI/ml (Xenetix, Guerbet, Roissy, France) was administered with an automated injector at a flow rate of 4 mL/sec. There was systematically a 3D MIP reconstruction (Maximum Intensity Projection) and a 2D multiplanar reconstruction (MPR). CTA data were transferred to a computer workstation (Advantage Workstation, AW 4.6; GE Medical Systems) for analysis. The reformatted 1.25-mm axial images, multiplanar reformats and Vessel Analysis software (GE Healthcare, Milwaukee, WI, USA) were used to determine the grade of stenosis. The normal diameter of the artery was measured proximal to the stenosis. The patient’s referring doctor ordered CTA at his or her discretion. CTA, used as gold standard, was performed to detect luminal arterial stenosis in each patient in our facility. Significant stenosis (≥ 50% of the diameter) at each artery level (aorta, common iliac artery, external iliac artery, common femoral artery, superficial femoral artery, popliteal artery on both sides) were reported by two blinded radiologists (AP and DB) who were unaware of the results of the exercise appointment. In case of variability higher than 10% for ≥ 50% stenosis between the two radiologists, a new interpretation was performed with both. The percent stenosis was calculated as follows by each physician: 100 x [1 –(diameter of the lumen at the site of the stenosis/diameter of the normal lumen)]. Finally, the degree of stenosis at each artery level used for the statistical analysis was calculated as the mean of the quantification performed by both physicians or in the case of a third interpretation, a third measurement was used.

### Statistical analyses

#### Sample size calculation

A sample size of 26 was estimated with 13 having the condition PAD (arterial stenosis ≥ 50%) and a sample size of 13 without the condition. An 80% power and 0.05% significance level were used to show a significant difference when comparing the area under the ROC curve (AUC) to a reference value for discrete response when the AUC under the null hypothesis is 0.500 and the AUC under the alternative hypothesis is 0.800.

#### Data analysis

Results are expressed as mean ± standard deviation in the case of normal distribution (Shapiro-Wilk test) or in median [25^th^ centile; 75^th^ centile] in the other cases. Receiving Operating Characteristic curves were used to study the relationship among: i) ABI and degrees of arterial stenosis; post-exercise ABI and degrees of arterial stenosis; iii) post-exercise ABI decrease and degrees of arterial stenosis; iv) post-exercise pressure decrease and degrees of arterial stenosis; and v) TcPO2 values (DROP) and degrees of arterial stenosis assessed using CTA.[8] The minimal DROP value at each calf site was used for the analysis. The DROP was the combination of the right and left distal DROP values. ROC curve analysis is based on calculating the sensitivity and specificity of a test for each value of the studied variable in the diagnosis of a disease state. An area under the curve (AUC) of 1.000 indicates perfect performance of the test, whereas an AUC of 0.500 indicates no discriminatory power. ROC curve determines a cutoff value that corresponds to the highest AUC for each test. Using ABI cut-off determined in the overall population, we performed a second ROC curve analysis to propose updated cut-offs for all exercise tests. Finally, additional analyses were performed to study if potential confounders could influence the conclusions. Indeed, for each test (with the corresponding cut off), we used multivariate logistic regression with a stepwise selection (p = 0.10) to adjust for potential confounders: sex, antihypertensive treatment (yes, no), diabetes (yes, no), dyslipidemia (yes, no) and tobacco habit (never, former, current). Statistical analyses were performed with MedCalc 12.6.1.0 software (MedCalc Software, Mariakerke, Belgium). For all statistical tests, a two-tailed probability level of p < 0.05 was used to indicate statistical significance.

## Results

**Fig 1:**
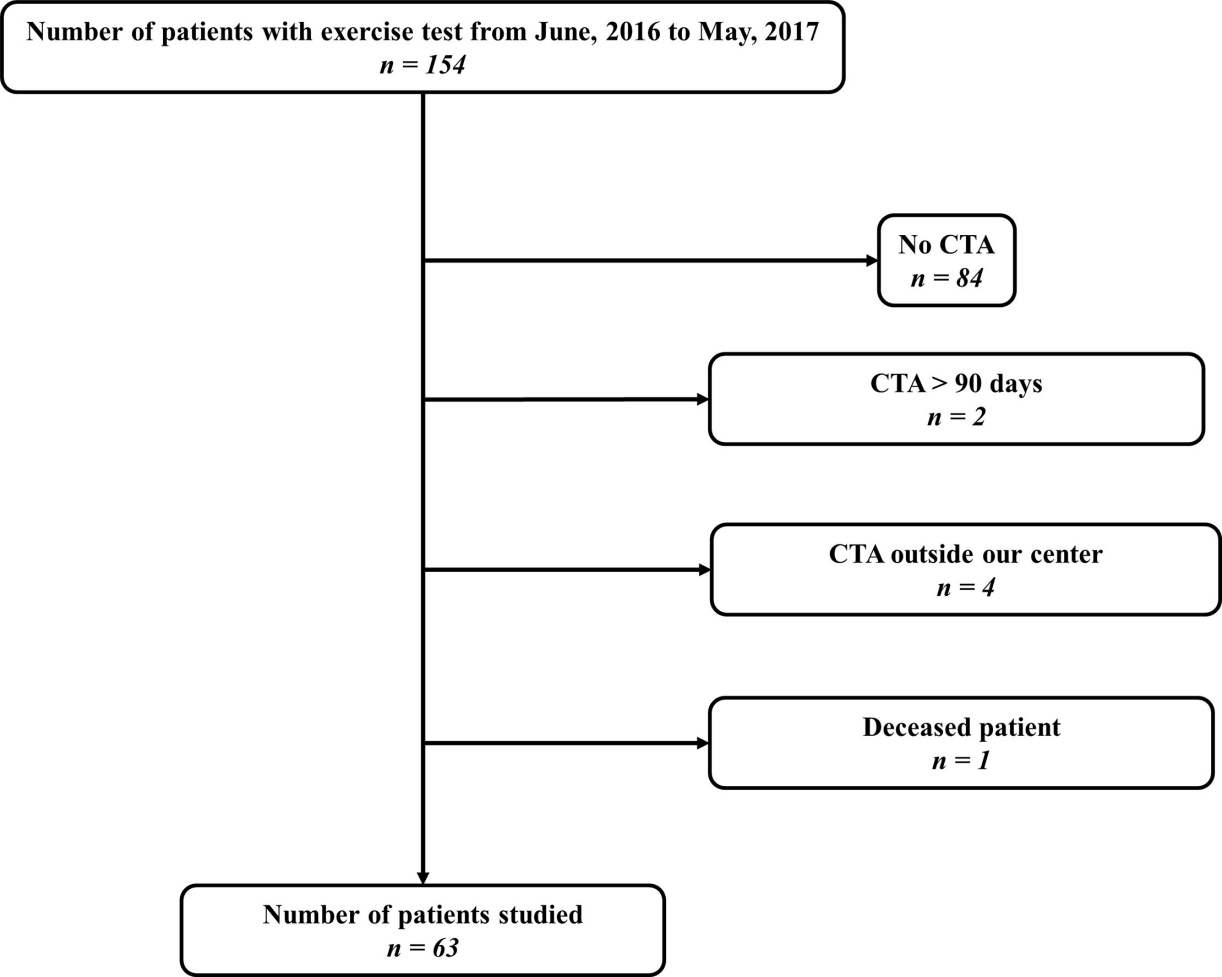
Flow chart. CTA, Computed tomography angiography.

Among 154 patients, 63 patients (83% men) met all criteria to be included in this prospective study (Fig 1). Study population characteristics and those from the subset of patients’ with at least one limb with an ABI > 0.91 (*n = 39 subjects*) are listed in Table 1.

**Table 1:** Population characteristics.

	Overall population (*n = 63*)	Subjects with at least one limb with an ABI > 0.91 (*n = 39*)
Age, years, mean (standard deviation)	61 (+/- 11)	61 (+/- 11)
Men, n (%)	52 (83)	30 (70)
Body mass index, kg/m^2^, mean (standard deviation)	27.1 (+/- 5.5)	26.7 (+/- 5.7)
Maximal walking distance self-reported, meter, median (25^th^ percentile, 75^th^ percentile)	400 (200–850)	500 (200–1000)
Ankle-brachial index, mean, (standard deviation)	0,87 (+/- 0.28)	1,09 (+/- 0.18)
Smoker (current smoker), n (%)	24 (46)	13 (33)
History of smoking, n (%)	29 (38)	20 (51)
Hypercholesterolemia, n (%)	45 (71)	25 (64)
Diabetes mellitus type 2, n (%)	16 (25)	8 (21)
Diabetes mellitus type 1, n (%)	0 (0)	0 (0)
Peripheral artery vascular revascularization*, n (%)	41 (65)	12 (31)
Hypertension, n (%)	42 (67)	26 (67)
Systolic blood pressure, mean, (standard deviation) (mmHg)	145 (+/-22)	146 (+/-19)
Diastolic blood pressure, mean, (standard deviation) (mmHg)	75 (+/-10)	75 (+/-10)
Coronary artery disease, n (%)	16 (25)	11 (28)
Prior stroke, n (%)	10 (16)	7 (18)
Statins, n (%)	34 (54)	23 (59)
Antihypertensive treatment, n (%)	46 (73)	27 (69)
Diuretics, n (%)	19 (30)	12 (31)
Angiotensin Converting Enzyme Inhibitors, n (%)	24 (38)	16 (41)
Angiotensin II receptor antagonists, n (%)	11 (18)	5 (13)
Calcium channel blockers, n (%)	23 (37)	11 (28)
Betablockers, n (%)	19 (30)	14 (36)
Antiplatelets, n (%)	54 (86)	31 (80)
Oral anti-diabetics treatment, n (%)	15 (24)	7 (18)

ABI, Ankle-brachial index

* Peripheral artery = Vascular surgery from aorta and/or more distal iliac or leg arteries.

Table 2 describes the prevalence and the anatomic level of stenosis ≥ 50% detected with CTA in our study population.

**Table 2:** Prevalence and anatomic level of stenosis ≥ 50% in either limb.

	Prevalence of stenosis ≥ 50% in either limb with an ABI > 0.91 (*n = 60*)	Prevalence of stenosis ≥ 50% in either limb among the overall population (*n = 126*)
Isolated aorto-iliac (aorta, common iliac artery and external iliac artery), n (%)	10 (17)	22 (17)
Isolated femoropopliteal (common femoral artery, superficial femoral artery, popliteal artery), n (%)	8 (13)	31 (25)
Aorto-iliac and Femoropopliteal, n (%)	3 (5)	26 (21)
Absence of aorto-iliac and femoropopliteal stenosis, n (%)	39 (65)	47 (37)

ABI, Ankle-brachial index.

**Fig 2:**
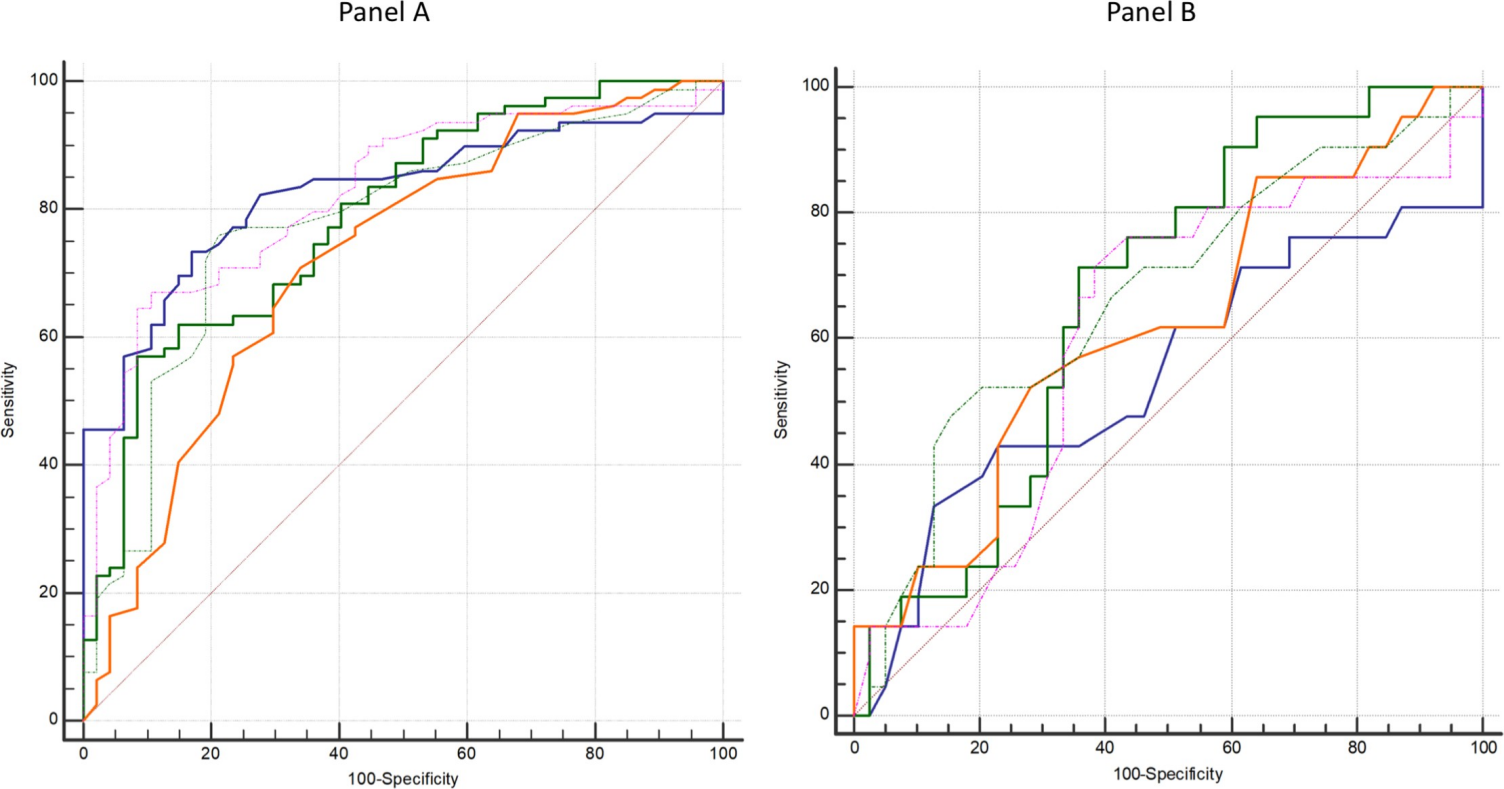
**ROC curves of the different tests to diagnose arterial stenosis ≥ 50% in the overall population (*n = 126 limbs*)** (A) and in the population with an ABI > 0.91 (*n = 60 limbs*) (B). Blue curve is ABI; Green curve post exercise ABI decrease; Orange post-exercise ankle pressure decrease; Green curve (incomplete line) exercise TcPO2 (DROP); Pink curve: post-exercise ABI.

Fig 2 (panel A) shows the ROC curves analysis, for the overall population (*n = 126 limbs*), that allows to determine a cutoff value that corresponds to the highest AUC for each test. Table 3 shows for the overall population, test characteristics (ABI, post-exercise ABI, post-exercise ABI decrease from ABI; post-exercise ankle pressure decrease from resting ankle pressure, DROP) to detect arterial stenosis ≥ 50%.

**Table 3:** Test characteristics for identifying stenosis ≥ 50% in any limb from aorta till popliteal artery in the overall population (*n = 126 limbs*).

Population (*n = 63*) Limbs (*n = 126*)	Cutoff	Sensitivity (95% CI)	Specificity (95% CI)	Positive predictive value (95% CI)	Negative predictive value (95% CI)	Accuracy (95% CI)	Area under curve (95% CI)	p-Value
ABI	≤ 0.91	73% [62–83]	83% [69–92]	88% [79–93]	65% [56–73]	77% [69–84]	0.83 [0.75–0.89]	<0.01
Post-exercise ABI	≤ 0.52	67% [56–77]	89% [77–97]	91% [82–96]	62% [54–69]	75% [67–83]	0.82 [0.75–0.87]	<0.01
Post-exercise ABI decrease	≥ 43%	57% [45–68]	89% [77–97]	90% [79–96]	55% [49–62]	69% [60–77]	0.79 [0.71–0.86]	<0.01
Post-exercise ankle pressure decrease	≥ 20 mmHg	71% [60–81]	66% [51–79]	78% [70–84]	57% [48–67]	69% [60–77]	0.72 [0.63–0.79]	<0.01
Exercise-TcPO2 (Distal DROP)	≤ -15 mmHg	76% [65–85]	79% [64–89]	86% [77–91]	66% [56–75]	77% [69–84]	0.78 [0.69–0.85]	<0.01

ABI, Ankle-brachial index; TcPO2, Transcutaneous oxygen pressure measurements.; DROP, Delta from rest oxygen pressure; CI: Confidence Interval.

For the overall population, all tests (ABI, post-exercise ABI, post-exercise ABI decrease, post-exercise pressure decrease and DROP) were statistically significant to detect arterial stenosis ≥ 50%. The test with the highest AUCs to detect arterial stenosis ≥ 50% was the ABI (Table 3).

Fig 2 (panel B) shows the ROC curves analysis, for the population with an ABI > 0.91 (*n = 60 limbs*), that allows to determine for each test a cutoff value that corresponds to the highest AUC. Table 4 shows exercise test characteristics of each exercise test in the population with an ABI > 0.91. In these subsets of patients, the AUCs of post-exercise ABI and post-exercise ankle pressure decrease ≥ 20mmHg were not statistically significant to detect arterial stenosis whereas statistical significances were obtained for post-exercise ABI decrease, and DROP with similar AUCs.

**Table 4:** Exercise test characteristics for identifying stenosis ≥ 50% in any limb among the 60 limbs with normal ABIs (> 0.91).

Population (*n = 39*) Limbs (*n = 60*)	Cutoff	Sensitivity (95% CI)	Specificity (95% CI)	Positive predictive value (95% CI)	Negative predictive value (95% CI)	Accuracy (95% CI)	Area under curve (95% CI)	p-Value
Post-exercise ABI	< 0.90	71% [48–89%]	62% [45–77%]	50% [38–62%]	80% [66–89%]	65% [52–77]	0.61 [0.47–0.73]	0.19
Post-exercise ABI decrease	≥ 18.5%	71% [48–89%]	64% [47–79%]	52% [39%-64%]	81% [67%-90%]	67% [53–78]	0.67 [0.53–0.78]	0.02
Post-exercise ankle pressure decrease	≥ 20 mmHg	52% [30–74]	72% [55–85]	50% [34–66]	74% [63–82]	65% [52–77]	0.68 [0.55–0.80]	0.12
Exercise-TcPO2 (Distal DROP)	≤ -15 mmHg	48% [26–70%]	85% [70–94%]	63% [41–80%]	75% [59–83%]	72% [59–83]	0.67 [0.53–0.78]	0.03

ABI, Ankle-brachial index; TcPO2, Transcutaneous oxygen pressure measurements; DROP, Delta from rest oxygen pressure; CI: Confidence Interval.

Supplemental population characteristics for the both treadmill tests are listed in S1 Table. Test characteristics of the post-exercise AHA criteria in our population are listed in S2 Table.

After multivariate logistic regression with a stepwise selection to adjust for potential confounders: sex, antihypertensive treatment, diabetes, dyslipidemia, and tobacco habit (never, former, current), our criteria for each exercise test was not modified since none of the covariates mentioned above were included in the final model (S3 and S4 Tables).

## Discussion

This study is the first to simultaneously determine the sensitivities and specificities of ABI and multiple exercise tests to detect arterial stenosis ≥ 50% assessed by CTA. We confirm that ABI has a similar AUC to detect arterial stenosis as exercise testing and show that the criteria defined in an overall population (all ABI values) cannot be used in patients with an ABI > 0.91.

The AHA guidelines and recent PAD guidelines recommend an ABI as a first line tool to detect PAD.[10,15] Our results confirm that even in patients with exertional limb symptoms and suspected of having a PAD, post-exercise ABI and exercise-TcPO2 testing have similar AUCs as the ABI. Our results are similar to Ouriel *et al*. showing that ABI has a similar AUC as post-exercise measurements.[5] From a clinical point of view, it reinforces the ABI as a first line test in patients with exertional limb pain suspected of having a PAD.[16]

According to the AHA, a post-exercise ABI decrease of > 20% from the resting value or post-exercise pressure decrease from resting value of > 30mm Hg are recommended to confirm the diagnose PAD (Class IIa; Level of Evidence A).[5,17,18] These criteria have been suggested by Ouriel et al. and Laing and Greenhalgh.[5,17] In both studies, these criteria were defined in patients without a defined ABI. Our results show that a cut-off defined in an overall population cannot be used in a population with a specific ABI value. Indeed, to detect an arterial stenosis ≥ 50%, the post-exercise ABI decrease is ≥ 43% in the overall population whereas the cut-off is ≥ 18.5% in the subset of patients with an ABI > 0.91. The higher decrease of post-exercise ABI in a population containing patients with an abnormal ABI is not surprising and was suggested by the papers from Ouriel *et al*. and Laing and Greenhalgh that showed that the more patients are diseased the more the decrease of the post-exercise ABI or ankle pressure are.[5,17]

Our study suggests updated post-exercise ABI criterion and has several strengths when compared to previous studies.[5,17] First, arterial stenosis quantification was objectively performed when compared to the study conducted by Ouriel *et al*. in which no degree of stenosis was reported.(5] Second, in the present study, the subjects’ characteristics are well-defined with only patients with exertional limb pain included whereas in the Ouriel *et al*. paper, patients with exertional limb pain and patients with rest pains, ulcers and gangrene were studied.[5] Third, the tests used by Ouriel *et al*. (2.4 km/h and 7% grade up to pain) and by Laing and Greenhalgh (4 km/h and 10% during 1 minute) are very specific and are rarely performed. In the current study we used a treadmill speed and incline (3.2km/h and 10%) performed more widely as a clinical routine.[5,19]

Another interesting point is that it is the first time that exercise-TcPO2 and post-exercise pressure or post-exercise ABI measurements have been performed in the same study. In the subset of patients with an ABI > 0.91, this study finds that AUCs were similar between post-exercise ABI decrease (0.67 [0.53–0.78]) and the DROP (0.67 [0.53–0.78]) meaning that either technique can be used to detect PAD. Due to time and technical constraints when performing exercise-TcPO2, our study suggests that post-exercise pressure measurement should be performed first to diagnose PAD in patients with an ABI > 0.91 and suspected of having a PAD. However it has been shown that exercise-TcPO2 is useful in the case of patients with proximal claudication, patients with non or poorly compressible arteries (ABI > 1.40) or in the case of suspected exercise induced hypoxemia.[20–24]

A recent study published by Aday *et al*. in 2018 (*n = 199 subjects*) suggests that post-exercise ABI criteria < 0.90 has a statistically significant sensitivity and specificity higher than the post-exercise AHA criteria.[25] In our study, the post-exercise ABI criterion is statistically not significant, probably due to a lack of power of our study (n = 39 subjects). Another study should be performed to compare post-exercise ABI criterion < 0.90 and post-exercise ABI decrease ≥ 18.5% or a combination of them to diagnose PAD.

Finally, the current study confirms that a DROP cut-off of -15mmHg is accurate to diagnose arterial stenosis in patients suspected of PAD.[7,8] It is important to note that the AUCs are not high in this study and a fair question remains; what is the optimal treadmill workload to be used for patients suspected of having a PAD?

### Limits

Our study has several limitations. First, among our population of 154 patients, 63 patients have been included and 91 excluded, as shows in Fig 1. Among these 91 patients excluded, the absence of CTA (*n = 84*) was the main cause. Indeed, the realization of a CTA was done only if patients were strongly suspected of having a PAD. The use of the CTA was performed according to AHA recommendations.[10] Of interest, the population studied in the present study especially the patients with an ABI ≥ 0.91 is the population that required exercise test according to AHA recommendations.[10] Second, the different tests were not randomized. For all patients we began by performing ABI, then exercise-TcPO2 and finally post-exercise pressure measurements. It was not possible to randomize the tests due to appointment time constraints. Third, it was not possible to assess the reproducibility of the different tests in the present study. However, the intra-observer coefficient of variation (CV) for the resting ABI in our vascular laboratory is 9.4% (typical error of the estimate is 0.06).[26,27] This variation is in the common range found in the literature.[10] The reproducibility of the exercise tests have been previously reported: Van Langen *et al*. found that the inter-observer variability of the ABI was 10% at rest and 21% post exercise.[28] The exercise TcPO2 reproducibility using the same protocol that we used has been reported by our colleagues as excellent.[29] Finally, the inter-observer agreement of multi-detector row CT scan is considered as excellent with k values (Cohen’s k statistic).[30] Fourth, we have shown in Table 2 that our population has heterogeneous anatomical lesions. Due to a lack of power, we could not study if any of the exercise tests have greater value for a specific anatomical location compares to another. Fifth, we did not analyze infrapopliteal arterial lesions since it was not possible to quantify the degree of stenosis accurately using CT scan. While a limitation from a physiologic perspective, from a clinical perspective focus therefore is placed on more commonly re-vascularized arterial locations. Sixth, we used an automatic blood pressure monitor to assess brachial blood pressure at rest and after exercise. We recognize that the AHA guidelines recommend performing all pressure measurements with a hand-held Doppler at rest.[10] However, our objective was that proposed criteria are widely applicable in clinical practice, where in most facilities; only one person is available to perform the measurement. Indeed, Gardner and Montgomery have shown at rest, that brachial systolic blood pressure was not significantly different among the Doppler and oscillometric methods.[31] Seventh, it was possible to compare neither the accuracy of the different exercise tests to diagnose arterial stenosis ≥ 50% nor difference of cut-off between men and women due to the small sample size. These points remain to be studied.

## Conclusion

Our study shows that ABI is as accurate as exercise testing (similar AUCs) in patients with exertional limb pain. In patients with an ABI > 0.91, we suggest cut off values of post-exercise ABI decrease ≥ 18.5%, or distal DROP ≤ -15mmHg, which have similar AUCs, to detect PAD. An external validation of these criteria seems important to confirm our findings.

## Supporting information

S1 Table Supplemental population characteristics for both treadmill tests. ABI, Ankle-brachial index.
(DOCX)

S2 Table Performance of AHA exercise criteria for identifying stenosis ≥ 50% in any limb among the 60 limbs with normal ABIs (> 0.91). ABI, Ankle-brachial index.
(DOCX)

S3 Table Association between test for identifying and stenosis ≥ 50% in any limb from aorta till popliteal artery in the overall population (*n = 126 limbs*) adjusted for sex, antihypertensive treatment, dyslipidemia, diabetes, tobacco. OR: Odds ratio; ABI: Ankle Brachial Index, TcPO2, Transcutaneous oxygen pressure measurements. DROP, Delta from rest oxygen pressure.
(DOCX)

S4 Table Association between test for identifying and stenosis ≥ 50% in any limb among the 60 limbs with normal ABIs (> 0.91) adjusted for sex, antihypertensive treatment, dyslipidemia, diabetes, tobacco. OR: Odds ratio; ABI: Ankle Brachial Index, TcPO2, Transcutaneous oxygen pressure measurements. DROP, Delta from rest oxygen pressure.
(DOCX)
