# Supplementary material for: Exercise testing criteria to diagnose lower extremity peripheral artery disease assessed by computed-tomography angiography
Source: PLoS One. 2019 Jun 27;14(6):e0219082. doi: 10.1371/journal.pone.0219082 (PMC6597112; doi:10.1371/journal.pone.0219082)
Supplement: S1 Table — ABI, Ankle-brachial index. (DOCX) [file pone.0219082.s001.docx]

**S1 Table. Supplemental population characteristics for the both treadmill tests.**

|  | Overall population (*n = 63*) | Subjects with at least one limb with an ABI > 0,91 (*n = 39*) |
| --- | --- | --- |
| Heart rate before the first test (exercise TcPO2 test), beat per minute, mean (95% CI) | 83 [78-88]* | 76 [71-81]* |
| Heart rate before the second test (post-exercise ABI test), beat per minute, mean (95% CI) | 80 [76-84]* | 80 (74-86]* |
| Maximal treadmill walking distance (TcPO2 test), meter, median (25th centile, 75th centile) | 218 (140-482) ** | 218 (139-585) ** |
| Maximal treadmill walking distance (post-exercise ABI test), meter, median (25th centile, 75th centile) | 215 (125-363)** | 185 (117-538)** |
| Indication of the treadmill test |  |  |
| Intermittent claudication, n (%) | 61 (97) | 37 (95) |
| Muscle fatigability (legs), n (%) | 2 (3) | 2 (5) |
| Cause of stopping for both treadmill tests | *n = 126* | *n = 78* |
| Limb or buttock pain, n (%) | 105 (83) | 58 (74) |
| Exhaustion, n (%) | 4 (3) | 4 (5) |
| Dyspnea, n (%) | 6 (5) | 6 (8) |
| No Stop, n (%) | 11 (9) | 10 (13) |

* There wasn't a statistical difference between the means of heart rate before exercise TcPO2 and post-exercise ABI tests (p<0.05).** There was a statistical difference between the means of the maximal treadmill walking distance of exercise TcP02 and post-exercise ABI tests (p<0.05). ABI, Ankle-brachial index. TcPO2, Transcutaneous oxygen pressure measurements. DROP, Delta from rest oxygen pressure; CI confidence interval.
