# Supplementary material for: Exercise testing criteria to diagnose lower extremity peripheral artery disease assessed by computed-tomography angiography
Source: PLoS One. 2019 Jun 27;14(6):e0219082. doi: 10.1371/journal.pone.0219082 (PMC6597112; doi:10.1371/journal.pone.0219082)
Supplement: S2 Table — ABI, Ankle-brachial index. (DOCX) [file pone.0219082.s002.docx]

**S2 Table 2. Performance of AHA exercise criteria for identifying stenosis ≥ 50% in any limb among the 60 limbs with normal ABIs (> 0.91).**

|  | Cutoff | Sensitivity (95% CI) | Specificity (95% CI) | Positive predictive value (95% CI) | Negative predictive value (95% CI) | Accuracy (95% CI) |
| --- | --- | --- | --- | --- | --- | --- |
| Post-exercise ABI decrease | > 20% | 62% [38-82] | 64% [47-79] | 48% [35-61] | 76% [63-85] | 63% [50-75] |
| Post-exercise ankle pressure decrease | >30 mmHg | 24% [8-47] | 82% [67-94] | 42% [21-67] | 67% [60-73] | 62% [48-74] |

ABI, Ankle-brachial index; CI confidence interval.
