# Supplementary material for: Exercise testing criteria to diagnose lower extremity peripheral artery disease assessed by computed-tomography angiography
Source: PLoS One. 2019 Jun 27;14(6):e0219082. doi: 10.1371/journal.pone.0219082 (PMC6597112; doi:10.1371/journal.pone.0219082)
Supplement: S3 Table — OR: Odds ratio; ABI: Ankle Brachial Index, TcPO2, Transcutaneous oxygen pressure measurements. DROP, Delta from rest oxygen pressure. (DOCX) [file pone.0219082.s003.docx]

**S3 Table. Association between test for identifying and stenosis ≥ 50% in any limb from aorta till popliteal artery in the overall population (*n = 126 limbs*) adjusted for sex, antihypertensive treatment, dyslipidemia, diabetes, tobacco.**

|  | Cutoff | OR [CI95%] |
| --- | --- | --- |
| ABI | ≤ 0.91 | 13.09 [5.14 ; 33.36] |
| Post-exercise ABI | ≤ 0.52 | 17.23 [6.06 ; 48.41] |
| Post-exercise ABI decrease | ≥ 43% | 14.22 [4.65 ; 43.48] |
| Post-exercise ankle pressure decrease | ≥ 20 mmHg | 3.81 [1.78 ; 8.18] |
| Exercise-TcPO2 (Distal DROP) | ≤ -15 mmHg | 11.68 [4.90 ; 27.83] |

OR: Odds ratio; ABI: Ankle Brachial Index, TcPO2, Transcutaneous oxygen pressure measurements. DROP, Delta from rest oxygen pressure.
