# Supplementary material for: Exercise testing criteria to diagnose lower extremity peripheral artery disease assessed by computed-tomography angiography
Source: PLoS One. 2019 Jun 27;14(6):e0219082. doi: 10.1371/journal.pone.0219082 (PMC6597112; doi:10.1371/journal.pone.0219082)
Supplement: S4 Table — OR: Odds ratio; ABI: Ankle Brachial Index, TcPO2, Transcutaneous oxygen pressure measurements. DROP, Delta from rest oxygen pressure. (DOCX) [file pone.0219082.s004.docx]

**S4 Table. Association between test for identifying and stenosis ≥ 50% in any limb among the 60 limbs with normal ABIs (> 0.91) adjusted for sex, antihypertensive treatment, dyslipidemia, diabetes, tobacco.**

|  | Cutoff | OR [CI95%] |
| --- | --- | --- |
| Post-exercise ABI | < 0.90 | 3.79 [0.95 ; 23.58] |
| Post-exercise ABI decrease | ≥ 18.5% | 3.80 [1.04 ; 24.59] |
| Post-exercise ankle pressure decrease | ≥ 20 mmHg | 2.77 [0.98 ; 7.92] |
| Exercise-TcPO2 (Distal DROP) | ≤ -15 mmHg | 4.71 [1.50 ; 14.78] |

OR: Odds ratio; ABI: Ankle Brachial Index, TcPO2, Transcutaneous oxygen pressure measurements. DROP, Delta from rest oxygen pressure; CI: Confidence Interval.
